# Examining the Consequences of Neuropsychological Test Battery Changes on Measuring Cognitive Decline

**DOI:** 10.1093/geroni/igaf122.2132

**Published:** 2025-12-31

**Authors:** Ryan Andrews, Brandon Gavett, Natalia Gomes Goncalves, Emma Nichols, Kendra Sims, Douglas Tommet, Yingyan Wu, Dan Mungas

**Affiliations:** Columbia University, New York, New York, United States; University of California, Davis, Davis, California, United States; Universidade de São Paulo Medical School, São Paulo, Sao Paulo, Brazil; University of Southern California, Los Angeles, California, United States; Boston University, Boston, Massachusetts, United States; Brown University, Providence, Rhode Island, United States; University of California, Los Angeles, Los Angeles, California, United States; University of California, Davis, Davis, California, United States

## Abstract

Large, representative studies of older adults have collected longitudinal cognitive data, but cognitive batteries often change over time. When cognitive batteries change, changes in measurement properties may result in misleading findings about cognitive trajectories. Using the Health and Retirement Study (HRS) as an example, we used simulation methods to assess the magnitude of bias in estimating cognitive decline induced by changing test batteries over time. We first simulated true cognition values using non-linear models of cognitive change derived from four harmonized longitudinal cognitive aging studies. We then used item response theory (IRT) methods to simulate measured test results for different cognitive testing batteries, including the introduction of the Harmonized Cognitive Assessment Protocol (HCAP) for a subset of HRS participants. We then estimated blended cognitive trajectories, artificially introducing changes of the simulated test to measure cognition. To illustrate the impact of these changes, we used linear mixed-effects models to estimate 11-year cognitive trajectories, overall and by quartile of true decline. Using instruments with the highest measurement precision led to estimated cognitive trajectories that best matched the truth; however, estimated trajectories using less informative test versions did not deviate from the truth by an appreciable amount. As such, we conclude that in practice, changes in test batteries over time may not meaningfully affect estimates of cognitive decline when IRT scoring is used.

